# Negative and positive psychological experience of frontline nurses in combatting COVID‐19: A qualitative study

**DOI:** 10.1111/jonm.13481

**Published:** 2021-11-01

**Authors:** Xin Peng, Yi Yang, Ping Gao, Yi Ren, Deying Hu, Qin He

**Affiliations:** ^1^ Cancer Center, Union Hospital, Tongji Medical College Huazhong University of Science and Technology Wuhan China; ^2^ Department of the Wise Group Pathways Health Hamilton New Zealand; ^3^ Department of Nursing, Union Hospital, Tongji Medical College Huazhong University of Science and Technology Wuhan China; ^4^ Public Health Department, Union Hospital, Tongji Medical College Huazhong University of Science and Technology Wuhan China

**Keywords:** frontline nurses, novel corona virus pneumonia, psychological experience, qualitative research

## Abstract

**Aims:**

To qualitatively explore potential experience among frontline nurses who had been fighting against the COVID‐19 infection since the outbreak.

**Background:**

Disasters are often sudden and uncertain. Since the COVID‐19 outbreak in Wuhan city, local frontline nurses had been responsible for treatment of COVID‐19 for several months. Qualitative study was required to assess complex multi‐component psychological experiences among frontline nurses.

**Methods:**

Twenty local frontline nurses were recruited from a designated hospital of COVID‐19 treatment. We conducted semi‐structured interview using phenomenological method. Descriptive phenomenological method was applied for thematic analysis.

**Results:**

Twenty female frontline nurses (aged 24 to 43 years old) were interviewed. Two broader themes, negative and positive, were identified. Negative experience included refusal and helpless (refusal to work at frontline, shortage of confidence in working and helpless), fear and anxiety, excessive miss, and other health issues. Positive experience included improved interpersonal relationship, sublimation of personal faith and strength, changes in understanding meaning of life and new possibility.

**Conclusion:**

Both positive and negative psychological response were observed, which can provide evidence based clues for making essential strategies and policy.

**Implications for Nursing Management:**

Understand subjective experience of frontline nurses can establish evidence for development of effective psychological intervention. Nursing administrator should consider the nurses' psychological experience comprehensively to promote psychological growth and lower post‐traumatic psychological burden.

## 1 BACKGROUND

Since early December, 2019, the first coronavirus disease 2019 (COVID‐19) case was detected in Wuhan, China. The COVID‐19 had a basic reproduction number (R0) of 5.7 and required timely diagnosis and effective treatment to prevent progression and lower mortality (Jin et al., 2020; Sanche et al., 2020). Person‐to‐person transmission of the disease made the first‐wave outbreak spread quickly, and it had evolved into a global pandemic. Although countries have tried their best to implement effective restrictions and promote vaccination, the global epidemic was not still contained (Han et al., 2020). Some countries had experienced a second wave or even the third wave of COVID‐19 (Asrani et al., 2021). As of August, 2021, the COVID‐19 had infected more than 200,000,000 cases worldwide. The global pandemic might be a long lasting problem; it is worthwhile and beneficial to study COVID‐19 related topics, including topics triggered at the beginning of the outbreak.

Since the outbreak, the healthcare workers (HCWs) have been responsible for the frontline fight against the COVID‐19 for several months. Necessity and importance of psychological status among frontline nurses have been emphasized (Chen et al., 2020). Negative psychological status might prevent nurses from caring for patients with COVID‐19 (Joo & Liu, 2021). A systematic review including 16 quantitative articles found that the frontline HCWs suffered from high prevalence of post‐traumatic stress symptoms (D'Ettorre et al., 2021). Qualitative studies reported psychological disturbances, powerless, depression and anxiety among frontline HCWs (Al Ghafri et al., 2020; Fang et al., 2021; Liu et al., 2020a). Higher anxiety and stress were observed among frontline HCWs who were female, married and had children (Celmece & Menekay, 2020; Huang et al., 2021). The conflict between family and work, increase in family care responsibilities, and constant worry that oneself and family members would be infected might explain this phenomenon.

While, coping with trauma events also might develop positive psychological experience (Meichenbaum, 2017). During the COVID‐19 pandemic, psychological resilience and effective coping strategies promoted positive psychological health outcomes among HCWs (Labrague, 2021). A quantitative study suggested frontline nurses experienced a moderate and high‐level post‐traumatic growth (PTG) (Peng et al., 2021). Both positive and negative elements in psychological responses were urgent and noteworthy that required comprehensive research. However, previous studies investigated either negative or positive experience separately; a comprehensive study on HCWs' mental health was inadequately understood. Qualitative study can give a comprehensive and in‐depth understanding of phenomenon and experience by exploring natures of research subjects and establishing relationship between them (Busetto et al., 2020). So qualitative method can equip us with a better tool to assess complex multi‐component psychological experiences among frontline nurses.

Disasters and crises on earth are often sudden and uncertain, including lessons Learned from COVID‐19 pandemic (Miller, 2020). At beginning of the outbreak as the first wave in Wuhan city, many hospitals were designated as the first‐line hospital for treatment of critical patients infected with COVID‐19. However, unavoidably increased workloads, shortage of personal protection equipment (PPE), uncertainty of curative treatment, risk of infection and death and impact of patients' pessimism had posed a great and significant threat on these frontline nurses (Huang et al., 2021). Some nurses in Wuhan city had been working at frontline since the city lockdown, and they might need to play the role of both a clinical nurse and a family caregiver. With the increase in support, they were required to quarantine in designated hotel after work, direct contact with families decreased. Compared with general population and nurses in the subsequent waves, more profound and comprehensive psychological experience might be observed among these frontline nurses. HCWs were limited and scarce resources to fight against current and future crisis; their initial response to the sudden disaster was worth studying to provide evidence for developing customized intervention and protect them from the next crisis.

In this study, a tertiary Grade A hospital of Wuhan city was selected, in which more than 5200 COVID‐19 patients and 30,000 fever patients were treated since the outbreak. We recruited 20 female frontline nurses from fever outpatient and isolation ward. The subjects were interviewed through semi‐structured interview. The objective of this qualitative study was to provide in‐depth insights into comprehensive experiences of frontline nurses from epidemic center. It would not only generate new knowledge regarding psychological development to sudden disaster but also establish evidence for development of effective psychological intervention.

## 2 METHODS

The consolidated criteria for reporting qualitative research (COREQ) guideline was implemented in this study for reporting methods and results structurally and clearly (Tong et al., 2007).

### 2.1 Study design and setting

To capture and describe the frontline nurses psychological experience compressively and veritably, we chose a qualitative method by in‐depth semi‐structured face‐to‐face interview. A descriptive phenomenological method was applied to understand aspects of experiences and generalizing the individual's reports in qualitative study of psychology (Englander, 2016; Matua & Van Der Wal, 2015).

Since January 23, 2020, the COVID‐19 outbreak was declared as a public health emergency and caused lockdown of the Wuhan city because of its high contagion and great uncertainty. Many local hospitals were reconstructed into designated hospitals for treating the COVID‐19. Many local nurses were arranged to support the treatment with limited source since the lockdown. A tertiary Grade A and designated hospital in Wuhan city was selected to recruit participants.

### 2.2 Ethical approval

This study was reviewed and approved by the Ethics Committee of the Union Hospital of Tongji Medical College, Huazhong University of Science and Technology (2020) Lunshenzi (0025), moreover, special approval was obtained from the new coronavirus pneumonia emergency in 2020 project (number 2020kfyXGYJ001). Written informed consensus was documented for each included participant and information confidentiality was guaranteed.

### 2.3 Study participants and data collection

Female frontline nurses who had been fighting against the COVID‐19 from the beginning of the pandemic to 15 March 2020 were recruited for face‐to‐face interview. Invitation was distributed in an online nursing resource group, and a convenience sampling method was adopted based on ease of availability. Inclusion criteria included the following: (1) registered nurse who had worked in this hospital at least 1 year; (2) the duration of frontline work was above 14 days; (3) nurses who had signed the consent form and volunteered to participate in this study. Nurses who was on sick leave and personal leave, was excluded from interview. Average of 16–24 interviews could reach the saturation in a qualitative study (Hennink et al., 2017). Interviews were conducted until thematic saturation was reached. Finally, 23 frontline nurses were connected, 3 participants declined the interview because of additional work. Twenty nurses, ageing from 24 to 43 years old, agreed to participate in the interviewed during March 17 to March 20.

P. X. was a female nurse in chief with experience in psychology and qualitative study and worked as the interviewer. Y. Y. was a female clinical nurse and worked as the facilitator. Y. Y. contacted the participants and re‐introduced the study purpose and arrangement before official interview. After treatment work, the interview was held in the hotel where frontline nurses were quarantined. To guarantee data saturation, each interview was conducted without time limit until no new topic was represented.

### 2.4 Contents of the interview

A semi‐structured guide with open‐ended questions was developed based on both existing literatures on nurses' psychological experience and nursing experts in this designated hospital (Bastos et al., 2018; Yuwanich et al., 2015). The guide included five questions: (1) What is your first reaction when you knew that you would be transferred to the frontline COVID‐19 ward? (2) What impressed you the most during the COVID‐19 epidemic? (3) What were your concerns when you were working on the frontline? (4) How did your family think about that you were going to the frontline? (5) How did you think about your future life after working on frontline? The Preliminary guide was piloted with one targeted frontline nurse and no change was made for the interview guide, the pilot interview was included in the final analysis.

### 2.5 Data analysis

All interviews were audio‐recorded and transcribed verbatim, and relevant memo notes were made during and after the interviews for further analysis. A seven‐step phenomenological analysis method developed by Colaizzi was carried out for content analysis (Abalos et al., 2016). After being familiar with the data, two researchers (P. X. and R. Y.) independently coded the transcript. Discrepancies between the two independent coders were discussed and refined with the third research (H. D. Y.) for consistency. Identified topics were discussed among all authors and then categorized through several iterations.

## 3 RESULTS

Basic characteristics about the 20 nurses were presented in Table 1. Negative and positive experiences were determined preliminary based on previous studies and coders' self‐experience.

**TABLE 1:** Demographic characteristics of the participants (*n* = 20)

ID	Education level	Nursing experience (year)	Working against COVID‐19(day)	Title	Married	Having child/children
A	Diploma	6	21	Nurse practitioner	Yes	Yes
B	Bachelor	6	21	Nurse practitioner	No	No
C	Bachelor	7	14	Nurse practitioner	Yes	Yes
D	Bachelor	13	14	Supervisor nurse	Yes	Yes
E	Bachelor	10	21	Supervisor nurse	Yes	Yes
F	Bachelor	17	21	Nurse practitioner	Yes	Yes
G	Bachelor	18	21	Supervisor nurse	Yes	Yes
H	Diploma	6	21	Nurse	No	No
I	Bachelor	6	21	Nurse	Yes	No
J	Bachelor	6	20	Nurse practitioner	Yes	No
K	Diploma	6	14	Nurse	No	No
L	Bachelor	10	14	Nurse practitioner	Yes	Yes
M	Bachelor	7	21	Nurse practitioner	Yes	Yes
N	Bachelor	7	21	Nurse practitioner	Yes	Yes
O	Bachelor	13	14	Supervisor nurse	Yes	Yes
P	Bachelor	25	21	Supervisor nurse	Yes	Yes
Q	Postgraduate	5	21	Nurse practitioner	Yes	No
R	Bachelor	8	14	Nurse practitioner	Yes	Yes
S	Bachelor	4	21	Nurse	No	No
T	Bachelor	3	21	Nurse	No	No

### 3.1 Theme 1: Negative experience

#### 3.1.1 Refusal and helpless

##### Refusal to work at frontline and helpless

COVID‐19 was a highly contagious disease. During the start of the COVID‐19 outbreak, no effective treatment was found, and the personal protective equipment was limited. However, these nurses had to work with great vulnerability and workload. Most nurses stated that they did not want to work in the fever clinic.

This disease was too terrible, I did not want to go to the fever clinic, I would resign if I has been assigned to the fever clinic. (Nurse C)

Another participant further added

I heard that there was a shortage of personal protective equipment (PPE) from the nurses who have worked in the fever clinic; I did not know how to protect herself from COVID‐19, thus she did not want to work there. (Nurse Z)

The COVID‐19 required timely diagnosis and effective treatment to prevent progression and lower mortality. However, in addition to supporting treatment, there was no effective curative treatment and nursing management at that time. These nurses felt helpless.

There was no specific medicine to treat COVID‐19, all I could do was watching the patients suffering. (Nurse M)

Nurse N further added a self‐example:

an elderly patient talked jokes with the nurses when I just came in to the clinic, however, after two days, I lost my ability of speech and motor. (Nurse N)

##### Shortage of confidence in working

The COVID‐19 outbreak was a public health emergency during the Chinese Spring Festival. There was a sudden and rapid rise in the number of suspected, confirmed and death cases. However, the number of respiratory nurse specialist was seriously insufficient, and other nurses are temporarily trained and arranged to participate in treatment.

it was the first time that she acknowledges this kind of disease, I was doubting about her nursing ability in terms of COVID‐19, and I concerned if I could keep up with the work pace of other colleagues. (Nurse Y)

when rescuing critically patients, I was not confident with my ability to manipulate the ventilator and high‐flow oxygen equipment, I was afraid of dragging down her colleagues. (Nurse B)

#### 3.1.2 Fear and anxiety

Almost all participants had experiencing fear and anxiety because they were working with real risk of being infected and in the special working environment with heavy equipment.

It was hard to sleep when she saw the news about the death of the doctors and nurses from infection. I had older people and children in her family, I was afraid of infecting her family members. (Nurse L)

Meanwhile, the continuous increase in infected cases suggested that the pandemic did not seem to end, which further deteriorated the condition.

Recently, the increase of patients in my clinic made me always think about these patients, even after getting off work. (Nurse A)

#### 3.1.3 Excessive miss

The majority of respondents had families and children. They were separated from their family at least 3 weeks. Participants expressed their miss and guilty to their families.

I had not seen my daughter for 2 months, my daughter called my auntie instead of mother when video chatting. My daughter could not recognize her now. (Nurse H)

Another nurse N further added

when I came to the frontline, my son was too young to walk. A few days before the interview, my mother sent me a video that my son could walk now, I felt so guilty for passing my son's growth. (Nurse N)

#### 3.1.4 Other health issues

Heavy protective equipment, high‐intensity workload and closed working environment, fear and anxiety caused them to suffer from various physical symptoms. Participants mentioned discomforts during their work and rest.

When I was working in the isolation ward, I always felt difficulty in breathing, weakness and dizziness. (Nurse Y)

Another participant suffered from circadian rhythm disturbance because of continuous work.

When I took rest at the hotel, I felt that the day and night were reversed, and suffered from appetite and sleeping problems. (Nurse B)

Another nurse reported lack of appetite caused by stress and anxiety, which led to abnormal weight loss.

Sometimes, I felt very hungry but had no appetite, and I had lost 5 kg in the last month. (Nurse Z)

### 3.2 Theme 2: positive experience

After experiencing the COVID‐19 epidemic, these frontline nurses expressed growth in this adversity.

#### 3.2.1 Improved interpersonal relationship

The collaborative practice within health‐care team was critical for treating the emergent COVID‐19. The participants would keep an open mind and establish mutual trust with colleagues and patients.

I was too competitive before, but now I knew I could ask for help when I got in trouble. My colleagues and nurse in chief always brought food for me. I felt that relationship between myself and colleges became closer since I worked in frontline. (Nurse Z)

Meanwhile, social support assisted HCWs to cope with the COVID‐19 by warm interpersonal relationship and the honour of being a nurse.

When I went to work, my neighbors gave smile to me every morning after knowing she was a nurse, the sense of approval and respect warmed me. (Nurse P)

Another nurse further added

‘thank you’ was a simple but beautiful word where the canteen lady delivered food for me, the security tested my body temperature every day and the volunteer drove me to work for free. (Nurse C)

#### 3.2.2 Sublimation of personal faith and strength

National medical aid mission was implemented by the government, and the whole country tried its best to fight against the pandemic. Powerful support from the country encouraged the participants greatly. They found that they were much stronger mentally and physically than they thought.

I felt scared and stressed about the frontline job at the beginning. However, I was no longer afraid after so many medical teams and colleagues from all over the country joined frontline work. I was proud of my contribution when my motherland encountered the disaster. (Nurse C)

#### 3.2.3 Change in understanding meaning of life

These frontline participants were at risk of infection and death every day, and they experienced both success and failure, both life and death during treatment of the COVID‐19.

I felt that nothing was more important than being alive after seeing life and death. Maintaining a positive attitude and being happy every day were the most important thing for me. (Nurse Z)

Participants expressed their emphasis on life.

Good health was the foundation of everything. I would cherish life more and pay more attention to my health than before. (Nurse Y)

My husband should slow down work pace and spend more time with me and our children. (Nurse R)

#### 3.2.4 New possibility

The epidemic was a new and unexpected life event, which prompted participants to re‐plan their future lives after reflecting on their past experiences.

After the epidemic, I wanted to try new things which I did not dare to do before, such as skydiving and bungee jumping. (Nurse E)

## 4 DISCUSSION

The COVID‐19 pandemic was an emergent public health event. In this qualitative study, the recruited nurses were local in the epicentre and had worked at frontline since the COVID‐19 outbreak in Wuhan city. Most of previous studies focused on HCWs' negative experiences, whereas their positive experiences were largely neglected. We documented lived experiences of these nurses and observed both positive and negative experiences among these frontline nurses.

The epidemic broke out suddenly, and the local health organisation might have insufficient professional experience with this new virus. In the early stages of the outbreak, frontline nurses were temporarily recruited and trained. High contagion of the COVID‐19 and uncertainty in treatment and prevention made them under considerable psychological stress. Meanwhile, the personal protective equipment was limited seriously, including N95/FFP2 respirators, face shields or goggles. These frontline nurses had to work under high risk of occupational exposure to the infection (Zhan, Anders, et al., 2020). The unprecedented challenge would make them hesitant to work and feel helpless in the high‐risk environment. Previous study in India reported that more than 70% HCWs hesitate to work during the COVID‐19 pandemic (Khasne et al., 2020). The majority of them were not specialist in communicable disease, and shortage of confidence in caring for patients was observed. In a previous qualitative study, nine frontline nurses who were recruited and had no infectious disease expertise also stated lacked confidence and felt powerless when caring for patients (Liu et al., 2020b).

Fear and anxiety were observed as negative experience, which had been described in many previous studies (Hu et al., 2020; Shen et al., 2021). Dr Wenliang Li, a key figure in the COVID‐19 epidemic in China and a hero for the HCWs, died on 7 February 2020, aged 33 years (Petersen et al., 2020). Other countries documented medical workers' infection and death on duty, even suicide (Montemurro, 2020; Sohrabi et al., 2020). Colleague's infection and death presented a direct and specific challenge to uninfected HCWs and may further worsen their mental situation. Excessive miss was a special but reasonable experience among these participants. With the management of COVID‐19 epidemic increased, the frontline staffs were arranged to live alone in the designated hotels. They worked with high‐density and long‐duration nursing workload, they also worried about family members who were quarantined at home, but they only could contact family members occasionally through online tools. Another study on HCWs also reported missing of direct contact with intimate people (Al Ghafri et al., 2020).

Negative physical issues were also discussed. Frontline nurses worked with heavy equipment and long shift‐time work during the COVID‐19 outbreak; they tended to presented burnout and emotional exhaustion (Wang et al., 2021; Zhang et al., 2020). A large sample and multicentre study reported that more than 50% frontier nurses suffered from insomnia during their frontline works (Zhan, Liu, et al., 2020). In this study, loss of appetite was observed. Previous study showed that nurses in Wuhan city had higher score in poor appetite or overeating than nurses in other cities (Ren et al., 2021). These health issues were associated with the negative psychological experiences and might further the conditions.

Although trauma caused negative experience, negative experiences also catalysed the development of positive change, while positive changes acted as a buffer against the negative experiences (Tedeschi et al., 2018). Coping with and adaption to trauma brought about the post‐traumatic growth (Wu et al., 2019). In a previous study, nurses demonstrated high positive growth when worked with war victims (Lev‐Wiesel et al., 2009). During treatment of COVID‐19 infected patients, nurses should trust and collaborate effectively with team members. After work, a social distance was also required, but video communication allowed them to be connected to their loved ones. Supports from colleagues, supervisors and families were the fundamental, which could encourage HCWs to fight with confidence, help HCWs control negative thoughts and relieve their psychological burden and miss (Blanco‐Donoso et al., 2020). Support from society gave a great and warm respect to HCWs' contribution and encouraged HCWs to seek mental help, which might increase HCWs' self‐efficacy and turn the trauma into positive growth (She et al., 2021).

After the outbreak, the Chinese government carried out ‘one province to aid one city’ medical aid by recruiting HCWs outside Hubei province who possessed advance clinical experience to assist the fight. By March 2020, there were 42,000 HCWs from 31 provinces participating in the national mission. Meanwhile, many companies donated sufficient materials to support frontline HCWs. The national solidarity promoted the development of strong sense of national pride and psychological affiliation with the country (David & Bar‐Tal, 2009). Collaboration with supportive human resources strengthened local HCWs' power. Many special welfares were given to these frontline HCWs, including anti‐epidemic memento and commendation conference organised by government, which affirmed their contrition to the combat against the COVID‐19 pandemic.

The COVID‐19 trauma was an unexpected event; HCWs faced the risk of infection and death during their work. The normal life had been drastically changed. After a trauma, finding meaning in a traumatic event made sense of what had happened. Previous study showed that finding the meaning of life could help recovery from a grief and improve post‐traumatic growth instead of post‐traumatic stress (de Jong et al., 2020). In our study, the experience made participants feel a sense of life's inherent value and hope to have a life worth living. Meanwhile, they embraced new possibility and make changes, which will enrich their future life. Similar findings were found in cares of children during the COVID‐19 pandemic (Stallard et al., 2021).

### 4.1 Implementation in nursing management and future crisis

Our study showed that both negative and positive experiences were present among HCWs. Because the COVID‐19 pandemic was still continuing, future crisis was possible. Essential strategies and policy were needed to support frontline HCWs: (1) accessibility to formal psychological support and intervention, which maintain frontline nurses' mental health, especially male HCWs (Huang et al., 2021); (2) establishment of comfortable communication and effective collaboration environment to reduce burnout; (3) assurance of support from family, colleague and society to improve professional identity; (4) reasonable shift arrangement and attractive nutrition to maintain frontline HCWs' physical power.

Our study had several limitations. Firstly, even though participants were given the opportunity to share their personal experiences of risk and resilience, only 20 frontline nurses were interviewed; possible selection bias might exist in the qualitative study. Secondly, only female nurses were included; gender asymmetry limited the generalizability of the study. Finally, only a single time point was applied; the change in psychological experience could not be observed.

## 5 CONCLUSION

Prevention and control of COVID‐19 was a special and heuristic mission related to human life and health. This study showed that these frontline nurses experienced both negative and positive experiences as response to the COVID‐19 outbreak. Nursing professionals were recommended to provide reasonable manpower arrangement and humanistic care to support development of psychological growth and to lower post‐traumatic psychological burden.

## FUNDING INFORMATION

The role of the 2020 COVID‐19 emergency special approval project of Huazhong University of Science and Technology in the design of the study and collection, analysis and interpretation of data and in writing the manuscript gave much support. The reference number is 2020kfyXGYJ001.

## CONFLICT OF INTEREST

The authors declare that they have no competing interests.

## AUTHOR CONTRIBUTIONS

P. X. initiated and conceived this research article, collected data and supported with the first‐line nurses' interviews, and participated in writing the original article. Y. Y., G. P. and R. Y. wrote the original article. D. Y. H. and Q. H. supervised the study and reviewed final manuscript.

## ETHICS STATEMENT

This study has been approved by the Ethics Committee of Drug Clinical Trials of Huazhong University of Science and Technology. It has been carried out in the city of Wuhan, located in the middle south of China, with the registration number 1900022422. The participants who have been involved in this study have signed the informed consent form before being included in the study.

## CONSENT FOR PUBLICATION

Not applicable.

## Data Availability

The data being used and analysed during the current study are available from the corresponding authors upon reasonable request.
